# Phase 3, Open-Label Multicenter Study of Sotatercept in Japanese Participants With Pulmonary Arterial Hypertension

**DOI:** 10.1016/j.jacasi.2025.12.009

**Published:** 2026-01-31

**Authors:** Hiromi Matsubara, Nobuhiro Tanabe, Takeshi Ogo, Kohtaro Abe, Takumi Inami, Yuko Maeda, Ichiro Arano, Masayoshi Shirakawa, Ryosuke Sakai, Alexandra G. Cornell, Sotatercept Study 020 Investigators, Ichizo Tsujino, Nobuhiro Yaoita, Toshihiko Sugiura, Nobuhiro Tanabe, Takumi Inami, Masaru Hatano, Takahiro Hiraide, Yuichi Tamura, Shiro Adachi, Takeshi Ogo, Yu Taniguchi, Hiromi Matsubara, Nobuhiro Tahara, Kohtaro Abe, Hiroshi Watanabe, Yoshihiro Dohi, Akiyoshi Hashimoto

**Affiliations:** a NHO Okayama Medical Center, Okayama, Japan; b Pulmonary Hypertension Center, Chibaken Saiseikai Narashino Hospital, Chiba, Japan; c Division of Pulmonary Circulation, Department of Cardiovascular Medicine, National Cerebral and Cardiovascular Center, Osaka, Japan; d Department of Cardiovascular Medicine Kyushu University Graduate School of Medical Sciences Fukuoka, Japan; e Department of Cardiovascular Medicine, Kyorin University School of Medicine, Tokyo, Japan; f MSD K.K., Tokyo, Japan; g Merck & Co, Inc, Rahway, New Jersey, USA

**Keywords:** 6-minute walk distance, activins, pulmonary arterial hypertension, pulmonary vascular resistance, sotatercept

## Abstract

**Background:**

Sotatercept is a first-in-class activin signaling inhibitor, developed for the treatment of pulmonary arterial hypertension (PAH).

**Objectives:**

This open-label single-arm, multicenter, phase 3 study was initiated to evaluate the efficacy and safety of sotatercept in Japanese participants with PAH.

**Methods:**

Forty-six adult Japanese participants with PAH on stable background therapy were enrolled and received subcutaneous sotatercept starting at 0.3 mg/kg with a target dose of 0.7 mg/kg administered every 3 weeks. The primary efficacy endpoint was the change from baseline in pulmonary vascular resistance at 24 weeks.

**Results:**

Of 46 participants, 29 (63%) were WHO functional class II and 17 (37%) were class III. At 24 weeks, pulmonary vascular resistance decreased 99.2 (95% CI: –129.6 to –68.4) dynes·s/cm^5^ and, 6-minute walk distance (key secondary endpoint) increased 41.8 (95% CI: 27.8-55.5) meters. There were 6 (13%) serious adverse events (AEs) and no discontinuations due to AEs in the primary period. Safety was consistent with the known safety profile for sotatercept with AEs that were clinically manageable.

**Conclusions:**

In this study of Japanese participants with PAH, improvements in pulmonary vascular resistance and 6-minute walk distance were observed, consistent with the pivotal phase 3 STELLAR (A Study of Sotatercept for the Treatment of Pulmonary Arterial Hypertension [MK-7692-003/A011-11]) trial. These data suggest that sotatercept may be an important treatment option for Japanese patients with PAH. (A Study of Sotatercept in Japanese Pulmonary Arterial Hypertension [PAH] Participants [MK-7962-020], NCT05818137; Japan Registry of Clinical Trials, jRCT2031230046)

Pulmonary arterial hypertension (PAH) is a serious condition that results in a progressive increase in pulmonary vascular resistance (PVR) with subsequent right ventricular dysfunction and is associated with premature death.^1^ In the absence of treatment, most patients with PAH succumb to heart failure.^2^ As of 2021, the global prevalence of PAH was estimated at 192,000 cases, with a notable sex disparity, as females constituted 62% of the cases. The estimated number of deaths was approximately 22,000 globally, reflecting an age-standardized mortality rate of 0.27 per 100,000.^3^ Additionally, the prevalence of PAH in different countries has been estimated to be between 10 and 52 cases per 1 million people.^4^, ^5^, ^6^, ^7^ Specifically in Japan, the estimated prevalence of PAH is 32 cases per 1 million people.^7^

The goal of standard-of-care pharmacologic therapy for PAH is focused on increasing blood flow through the pulmonary vasculature via pharmacologic manipulation of various pathways to relieve symptoms and slow clinical worsening of the disease. Pharmacologic agents such as endothelin receptor antagonists (ERAs), phosphodiesterase type 5 inhibitors, guanylate cyclase stimulators, and prostacyclin analogs/prostacyclin receptor agonists have been used to attain these objectives.^8^ In Japan, adherence to current medications has led to improvements in clinical endpoints such as PVR, which is associated with longer transplant-free survival,^9^^,^^10^ 6-minute walk distance (6MWD) showing improved exercise capacity, and N-terminal pro–B-type natriuretic peptide (NT-proBNP), suggesting better cardiac function and reduced heart strain.^7^ However, survival with current treatment paradigms remains unsatisfactory with an estimated 5-year survival of only approximately 55%.^11^ According to one study, the rate of mortality after 5 years was estimated to be 26% in PAH patients in Japan even though most PAH patients in the study received treatment with a combination of 2 or 3 PAH drugs including parenteral prostacyclin.^12^ Treatments that target the underlying pathophysiology of PAH and confer more significant improvements with disease-modifying effects are an unmet need.

Sotatercept is a recombinant fusion protein consisting of the crystallizable fragment domain of human immunoglobulin G linked to the extracellular domain of human activin receptor type IIA, which acts as a ligand trap for selected transforming growth factor-β superfamily members. Inhibition of these ligands by sotatercept is proposed to improve the balance between pro-proliferative and antiproliferative signaling. The phase 2 PULSAR (Sotatercept for the Treatment of Pulmonary Arterial Hypertension) trial showed significant improvements with sotatercept in the primary endpoint of PVR from baseline to week 24, along with improvements in secondary endpoints such as the 6MWD and World Health Organization (WHO) functional class assessments.^13^ Following this, the phase 3 randomized, placebo-controlled STELLAR (Phase 3 Trial of Sotatercept for Treatment of Pulmonary Arterial Hypertension) trial was conducted in a larger PAH population to further evaluate efficacy and safety of sotatercept.^14^ The STELLAR trial showed that treatment with sotatercept significantly increased 6MWD and improved WHO functional class at 24 weeks compared to placebo, thereby highlighting its potential to enhance exercise capacity and overall functional status in patients with PAH.^13^^,^^14^ The most commonly reported adverse events (AEs) in these studies for sotatercept participants were telangiectasia and epistaxis, which were mostly mild or moderate in intensity and did not lead to treatment discontinuation. Increased hemoglobin (Hb) and decreased platelet counts, known effects of sotatercept, were generally clinically manageable with monitoring and use of dose interruptions or reductions and were not associated with treatment discontinuations. Based on this research, sotatercept was approved by the Food and Drug Administration in the United States and by the European Medicine Agency in the European Union for the treatment of PAH. More recently, long-term extension data in the SOTERIA (A long-term follow-up study of sotatercept for treatment of pulmonary arterial hypertension: interim results of SOTERIA)) trial showed sustained improvements in clinical endpoints with no new safety signals^15^ and the phase 3 ZENITH (Sotatercept in Patients with Pulmonary Arterial Hypertension at High Risk for Death) trial showed that sotatercept significantly reduced the composite risk of death, lung transplantation, or hospitalization for worsening PAH in high-risk patients receiving background therapy.^16^ Additionally, the phase 3 HYPERION (Sotatercept for pulmonary arterial hypertension within the first year after diagnosis) trial showed a lower risk of clinical worsening with the addition of sotatercept to background therapy than placebo in patients who received a diagnosis of PAH <1 year earlier showing benefit of early intervention using sotatercept.^17^ Although sotatercept has been studied as a treatment for PAH in global trial populations, as yet, there has been no dedicated clinical trial evaluating sotatercept in an Asian population.

This open-label phase 3 study in Japanese participants with PAH evaluated the efficacy of sotatercept in improving PVR and to assess its safety and tolerability in this specific population. Given the limited treatment options and the unique genetic and environmental factors that may influence PAH in Japanese individuals, understanding the efficacy and safety profile of sotatercept in this specific population is of importance.

## Methods

### Study design

This study (MK-7962 [sotatercept] Protocol 020, NCT05818137; Japan Registry of Clinical Trials, jRCT2031230046) was initiated on May 10, 2023, and is ongoing. The primary analysis period has been completed as of the data cutoff date of March 12, 2024, encompassing 24 weeks of open-label sotatercept treatment for participants. The study was conducted across 17 clinical sites in Japan and adhered to the principles of Good Clinical Practice. The study protocols received approval from the institutional review boards at each participating clinical site (Supplemental Table 1). All participants provided written informed consent before their enrollment in the study.

**Figure 1:**
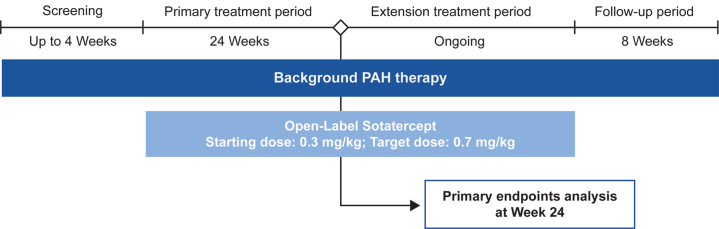
Study Design This open-label study included screening, primary treatment, extension treatment, and follow-up periods. The primary analysis was compete at week 24. PAH = pulmonary arterial hypertension.

This was a nonrandomized, noncontrolled, multicenter, open-label study examining the efficacy and safety of sotatercept in Japanese participants diagnosed with PAH. The study design included a screening period, a primary treatment period, an extension treatment period, and a follow-up period (Figure 1). The screening period lasted up to 4 weeks, during which eligible participants were assessed for inclusion based on specific criteria. The primary treatment period was 24 weeks in duration, during which participants received sotatercept at a starting dose of 0.3 mg/kg. The dose was subsequently increased to the target dose of 0.7 mg/kg. Throughout the study, participants were to continue their pre-existing background PAH therapies. Dose escalation was governed by predetermined protocols, and modifications (including potential dose delay, reduction, or discontinuation) were made based on both the guidance outlined in the study protocol and the clinical judgment of the investigators (Supplemental Figure 1).

After completing the primary treatment period, participants were given the option to enter the extension treatment period, when they may continue to receive sotatercept until regulatory approval was obtained or until they chose to discontinue participation. Participants who were deemed eligible and demonstrated proficiency in self-administration of the medication were to begin self-administration at home as early as week 33. These participants were to attend follow-up visits at their respective study sites every 4 visits after the initiation of self-administration.

The follow-up period, lasting at least 8 weeks, consisted of end-of-treatment and end-of-study visits. This period included participants who either decided not to continue in the extension treatment period or who discontinued the study medication before its completion.

### Participant selection

Participants were male and female adults 18 years of age and older, all of whom had a documented diagnosis of PAH confirmed through right heart catheterization. Participants were in one of the PAH subtypes: idiopathic PAH, heritable PAH, drug/toxin-induced PAH, PAH associated with connective tissue disease, or PAH linked to simple congenital systemic-to-pulmonary shunts at least 1-year post-repair. Additional criteria for inclusion were classification of PAH as either WHO functional class I or symptomatic functional class II-IV, a baseline right heart catheterization showing a PVR of ≥5 WU, and stability on background PAH therapies and diuretics for a minimum of 90 days before screening. Another requirement for inclusion was the ability to achieve a 6MWD between 150 and 500 meters, with both measurements needing to be within 15% of one another during screening.

Participants were excluded if diagnosed with pulmonary hypertension classified under groups 2, 3, 4, or 5 pulmonary hypertension, as well as certain subtypes of PAH such as PAH associated with HIV, portal hypertension, schistosomiasis, or those with significant venous/capillary involvement. Other exclusionary criteria were being on the lung transplant waiting list (unless their registration was within 12 months before screening), Hb levels above the sex-specific upper limit of normal at screening, or a platelet count below 50,000/mm^3^. Additionally, participants showing uncontrolled systemic hypertension (with sitting systolic blood pressure (BP) >160 mm Hg or sitting diastolic BP >100 mm Hg) or a baseline systolic BP <90 mm Hg at screening were not eligible to participate in the study.

### Efficacy measurements

The primary efficacy endpoint was the change in PVR at week 24. Secondary endpoints included changes in 6MWD, functional improvements as measured by WHO functional class, and NT-proBNP levels at week 24.

Also evaluated was multicomponent improvement, which included measurement of the proportion of participants achieving all 3 of the following: 1) improvement in 6MWD (increase ≥30 m); 2) improvement in NT-proBNP (decrease in NT-proBNP ≥30%) or maintenance/achievement of NT-proBNP level <300 pg/mL; and 3) improvement in WHO functional class or maintenance of WHO functional class I or II. Additional efficacy endpoints included hemodynamic parameters other than PVR (right atrial pressure, mean pulmonary artery pressure, mean pulmonary artery wedge pressure, mixed venous saturation of oxygen, and cardiac output at week 24) and time to clinical worsening (TTCW). TTCW was defined as the time from allocation to the first occurrence of any of the following: death; lung and/or heart transplantation; need to initiate rescue therapy with an approved PAH therapy or the need to increase the dose of parenteral prostacyclin by 10% or more; need for atrial septostomy; hospitalization for worsening of PAH (≥24 hours); deterioration of PAH defined by both of the following events occurring at any time, even if they began at different times, as compared to their baseline values: 1) worsened WHO functional class; and 2) decrease in 6MWD by ≥15% confirmed by 2 tests at least 4 hours apart, but no more than 1 week.

### Safety assessment

The primary safety objective was evaluation of safety and tolerability through the documentation of AEs and related discontinuations. Clinical and laboratory AEs and vital signs were monitored throughout the study. AEs were assessed by investigators for severity and relationship to the study medication. Serious adverse events (SAEs) and deaths were to be documented.

The safety assessments encompassed a variety of specific parameters, including event severity assessments, potential drug-related events, and changes in prespecified laboratory values and vital signs. Investigators classified AEs based on level of severity according to defined criteria which include major categories of mild, moderate, and severe events. SAEs were classified based on established definitions including the potential for hospitalization, significant disability, or any medical condition that poses an immediate risk to life.

### Available data

The analysis was based on all data collected during the primary treatment period and the extension treatment period through the data cutoff date of March 12, 2024.

### Statistical analysis

The efficacy analyses were based on the full analysis set population, defined as all participants who received at least 1 dose of study intervention. The estimate of the location parameter and the corresponding 95% CI based on the Hodges-Lehmann method was calculated for change from baseline in PVR at week 24. Change from baseline in 6MWD at week 24 and change from baseline in NT-proBNP at week 24 were evaluated in the same manner as PVR. The proportion of participants with improvement in WHO functional class at week 24 and the corresponding 95% CI based on the method of Clopper and Pearson were calculated. At the request of a reviewer, a post hoc analysis using a mixed effects quantile regression model was performed to take variability sources in effect estimates into consideration. The model included age, sex, baseline value, WHO functional class at baseline, and infusion prostacyclin use at baseline as fixed effect covariates and study center as a random effect.

Safety analyses were based on all participants as treated population, defined as all participants who received at least 1 dose of study intervention. The numbers and percentages of participants with at least one AE, drug-related AE, SAE, drug-related SAE, discontinuation from study intervention due to an AE, and an AE resulting in death, were summarized. The numbers and percentages of participants with telangiectasia (event of special interest) and other selected events including leukopenia, neutropenia (including febrile neutropenia), embryo-fetal toxicity, hepatic toxicity, cardiac events, thrombotic events, increased Hb, immunogenicity, thrombocytopenia, renal toxicity, bleeding events, increased blood pressure, or suppression of follicle stimulating hormone were also summarized. AEs were coded according to the MedDRA version 27.0.

The primary timepoint analysis was performed after all participants had completed week 24 or discontinued before week 24.

## Results

### Participants

**Figure 2:**
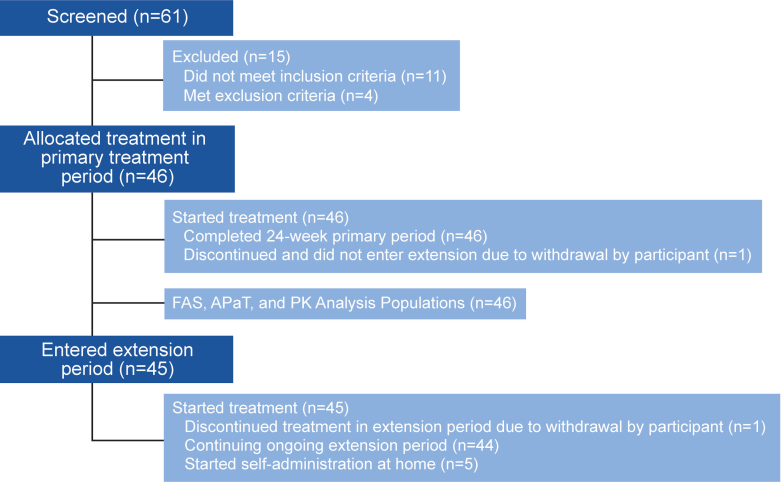
CONSORT Diagram There were 46 participants who initiated sotatercept treatment and completed the primary treatment period; 45 participants entered the extension period. APaT = all participants as treated; FAS = full analysis set; PK = pharmacokinetic.

A total of 61 participants were screened in this study and 46 were allocated to the study intervention across 15 study sites in Japan. All 46 allocated participants received at least 1 dose of study intervention, and all participants completed the primary treatment period (Figure 2); 45 participants entered the extension treatment period. Of these 45 participants, 1 discontinued the study intervention and the study due to withdrawal by subject during the extension treatment period, and 44 were continuing the study intervention and the study at the time of the data cutoff. Of 45 participants who entered the extension treatment period, 5 participants started self-administration at home at week 33 or later.

The mean age was 44.7 years, mean weight was 53.1 kg, and 74% of participants (n = 34 of 46 participants) were female. The median (Q1-Q3) time from PAH diagnosis to allocation was 10.6 (Q1-Q3: 4.0-18.7) years. The most common PAH subtypes were idiopathic PAH (54.3%, 25 participants), followed by heritable PAH (21.7%, 10 participants), PAH associated with simple, congenital systemic-to-pulmonary shunts at least 1 year following repair (13.0%, 6 participants), and PAH associated with connective tissue disease (10.9%, 5 participants). The proportion of participants with WHO functional class II was 63.0% (29 participants) and WHO functional class III was 37.0% (17 participants); no participants with WHO functional class I or IV were enrolled. There were more participants on background PAH triple therapy (93.5%, 43 participants) compared with double therapy (6.5%, 3 participants), and no participants used monotherapy at baseline. Parenteral prostacyclin therapy was used by 45.7% of participants (21 participants) at baseline (Table 1).

**Table 1:** Baseline Participant Characteristics

Participants in population	46 (100)
Sex	
Male	12 (26.1)
Female	34 (73.9)
Age, y	
Mean ± SD	44.7 ± 16.3
Range	20 to 80
Duration of PAH, y^a^	
Median (Q1-Q3)	10.6 (4.0-18.7)
Range	0.5-46.7
PAH diagnostic subgroup	
Idiopathic PAH	25 (54.3)
Heritable PAH	10 (21.7)
PAH associated with connective tissue disease	5 (10.9)
PAH associated with simple, congenital systemic-to-pulmonary shunts at least 1 year following repair	6 (13.0)
WHO functional class at baseline	
II	29 (63.0)
III	17 (37.0)
Background PAH therapy at baseline	
Monotherapy	0 (0.0)
Double therapy	3 (6.5)
Triple therapy	43 (93.5)
PVR at baseline, dynes·s/cm^5^	
Median (Q1-Q3)	536.0 (447.2-626.4)
≤800	43 (93.5)
>800	3 (6.5)
Prostacyclin infusion therapy at baseline	
Yes	21 (45.7)
No	25 (54.3)
Cardiac index at baseline, L/min/m^2^	
<2.5	8 (17.4) 38 (82.6)
≥2.5	
Cardiac output, L/min	4.8 ± 1.1
Pulmonary arterial wedge pressure, mm Hg	8.1 ± 3.0
Right atrial pressure, mm Hg	5.2 ± 2.9
Mixed venous oxygen saturation, %	70.5 ± 5.3
Mean pulmonary artery pressure, mm Hg	41.1 ± 9.0
6-Minute walk distance, m	404.0 ± 61.8
NT-proBNP, pg/mL	90.5 (45.0-147.0)

Values are n (%), mean ± SD, or median (Q1-Q3).

NT-proBNP = N-terminal pro–B-type natriuretic peptide; PAH = pulmonary arterial hypertension.

a The duration of PAH (years) was calculated as (date of treatment allocation – date of diagnosis of PAH)/365.25. If month is unknown, then set to January; if day is unknown, then set to the 15th of the month; if both month and day are unknown, then set to January 1st; if year is unknown, then do not impute.

### Efficacy

**Figure 3:**
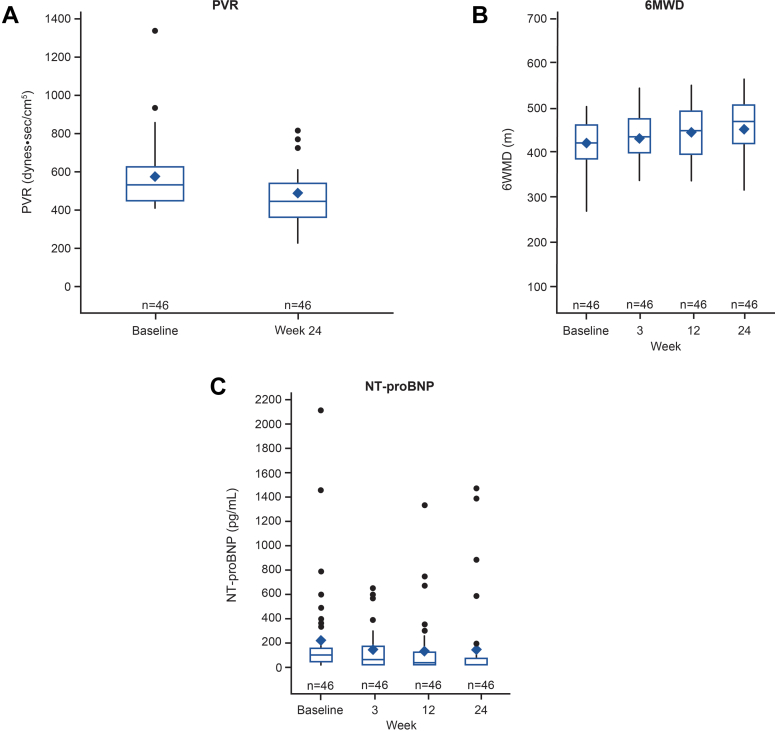
Efficacy Measures Over Time Boxplots depicting changes from baseline to end of primary treatment period in (A) pulmonary vascular resistance (PVR) (dynes·s/cm^5^), (B) 6-minute walk distance (6MWD) (meters), and (C) N-terminal pro–B-type natriuretic peptide (NT-proBNP) levels (pg/mL) in participants receiving sotatercept in addition to background therapy. Each boxplot shows the median, mean (filled diamond), interquartile range (IQR), with the whiskers extending to the minimum and maximum datapoints within 1.5 times IQR from the first and third quartiles. The observations falling outside of those limits are plotted individually. A trend of reduction in PVR and NT-proBNP levels, and improvement in 6MWD were observed up to 24 weeks after the start of additional treatment with sotatercept.

Sotatercept treatment resulted in a decrease in PVR at week 24 compared to baseline. The Hodges-Lehman location estimate for change from baseline in PVR at week 24 was –99.2 dynes·s/cm^5^ (95% CI: –129.6 to –68.4) (Table 2). The secondary endpoint of 6MWD at week 24 was supportive of the primary endpoint and showed that sotatercept treatment resulted in an increase in 6MWD at week 24 of 41.8 meters (95% CI: 27.8-55.5) compared to baseline. Additionally, sotatercept treatment resulted in a decrease in NT-proBNP at week 24 compared to baseline of –48.5 pg/mL (95% CI: –77.0 to –24.8) (Table 2). Observed values for these endpoints at baseline and at week 24 showed an improvement as well and are shown in Figure 3. The results from the post hoc analysis of mixed effects quantile regression were generally consistent with those from the Hodges-Lehmann method (Supplemental Table 2). Subgroup analyses for these endpoints showed that improvements were consistent across subgroups defined according to baseline characteristics including sex, PAH subtype, background PAH therapy, WHO functional class, PVR (≤800 vs >800 dynes·s/cm^5^), use of parenteral prostacyclin therapy, and cardiac index (<2.5 vs ≥2.5 L/min/m^2^) (Supplemental Figures 2 to 4).

**Table 2:** Summary of Efficacy (N = 46)

Treatment	Baseline		Week 24 Median	Change From Baseline at Week 24^a^	
	Median	Range		Range	Estimate (95% CI)
Change from baseline in PVR (dynes·s/cm^5^) at week 24	536.0	405.6-1,337.6	417.2	235.2-812.8	–99.2 –129.6 to –68.4
Change from baseline in 6MWD (m) at week 24	407.5	253.5-496.0	462.0	305.0-560.0	41.8 (27.8-55.5)
Change from baseline in NT-proBNP (pg/mL) at week 24	90.5	18.5-2,116.0	18.5	18.5-1,471.0	–48.5 –77.0 to –24.8

6MWD = 6-minute walk distance; PVR = pulmonary vascular resistance; other abbreviation as in Table 1.

a Based on the Hodges-Lehmann method. The primary approach was to exclude the data after addition, increase or substitute of background PAH therapy, but no participants modified the background PAH therapy during the primary treatment period and thus no data were excluded. Data cutoff was March 12, 2024.

**Figure 4:**
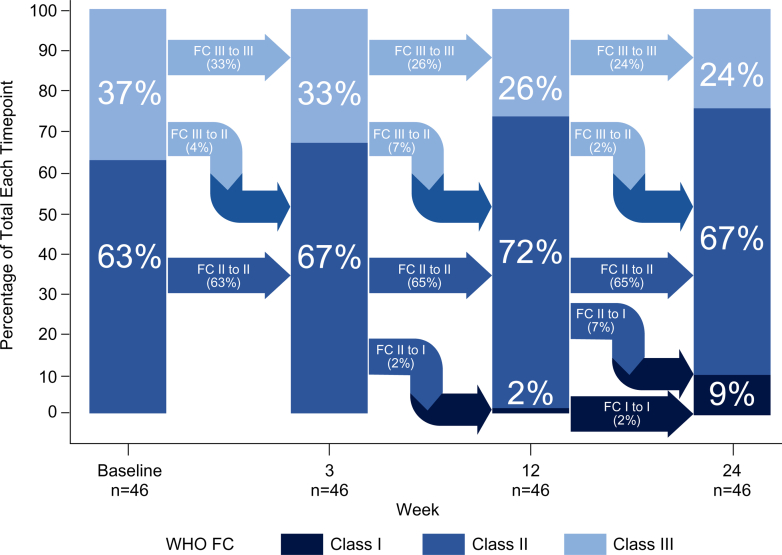
WHO Functional Class Over Time Stacked bar plots showing the proportion of participants in each WHO functional class (FC) at baseline and weeks 3, 12, and 24. A trend of improvement in WHO functional class was observed since the start of additional treatment with sotatercept.

The proportion of participants with improvement from baseline in WHO functional class at week 24 was 19.6% (9 participants, 95% CI: 9.4-33.9). Among these participants, 10.9% (5 participants) shifted from WHO functional class III to II, 6.5% (3 participants) shifted from WHO functional class II to I, and 2.2% (1 participant) shifted from WHO functional class III to I at week 24 (Figure 4).

Additional endpoints were supportive of these primary and secondary endpoints. The proportion of participants who met all 3 criteria of the multicomponent improvement endpoints was 50.0% (23 participants, 95% CI: 34.9-65.1) at week 24. Analyses of TTCW were performed using data up to the data cutoff (March 12, 2024). No participant died during this period; only 1 participant had ≥1 clinical worsening event at day 256 (hospitalization for worsening of PAH, followed by the need to increase the dose of parenteral prostacyclin by 10% or more) (Supplemental Figure 5). Regarding hemodynamic parameters at week 24, mean pulmonary artery pressure showed improvement from baseline whereas cardiac output, mean pulmonary arterial wedge pressure, right atrial pressure, venous saturation of oxygen, and cardiac index did not show any notable change (Supplemental Table 3).

### Safety and tolerability

There were 43 participants (93.5%) who experienced 1 or more AEs through the primary treatment period. The most common AEs (incidence ≥10%) were nasopharyngitis (30.4%, 14 participants), headache (21.7%, 10 participants), and increased Hb and epistaxis (17.4%, 8 participants each). The proportion and the number of participants with SAEs reported during the primary treatment period was 13.0% (6 participants). There were no SAEs that were observed in 2 or more participants. No SAEs were considered related to study treatment and no SAEs led to discontinuation of study intervention or withdrawal from the study (Table 3).

**Table 3:** Summary of Safety and Tolerability (N = 46)

Summary of AEs in primary treatment period	
≥1 AE	43 (93.5)
Serious AEs	6 (13.0)
Discontinuations due to AEs	0 (0.0)
Deaths	0 (0.0)
Drug-related AEs	27 (58.7)
Drug-related Serious AEs	0 (0.0)
Most common AEs (≥10%)	
Nasopharyngitis	14 (30.4)
Hb Increased	8 (17.4)
Headache	10 (21.7)
Epistaxis	8 (17.4)
AEs of interest (≥5%)	
Telangiectasia	3 (6.5)
Epistaxis	8 (17.4)
Rash	3 (6.5)
Hb Increased	8 (17.4)
Thrombocytopenia^a^	4 (8.7)

Values are n (%).

AE = adverse events; Hb = hemoglobin.

a Reported as thrombocytopenia or platelet count decreased.

Regarding the AE of special interest, telangiectasia was reported in 3 participants (6.5%). Other events of interest included bleeding. Bleeding events occurred in 13 participants; specifically, 8 participants reported epistaxis and the following bleeding-related events were reported by 1 participant each: anemia, contusion, gingival bleeding, subcutaneous hemorrhage, injection site bruising, and lower gastrointestinal hemorrhage. Increased Hb occurred in 8 participants. Thrombocytopenia occurred in 4 participants. All 3 (6.5%) telangiectasia, 6 (13.0%) of the bleeding events, all 8 (17.4%) increased Hb, and 2 (4.3%) thrombocytopenia events were considered related to study intervention by the investigator. Overall, minimal changes in Hb and platelet counts were observed with mean Hb levels increased from baseline by 1.1 g/dL at week 24, and the mean platelet count decreased from baseline by 0.8 × 10^9^/L at week 24.

Dose delays due to AEs occurred in 3 participants (6.5%) per-protocol dose modification guidelines (1 increased Hb and 2 thrombocytopenia cases). The AE of increased Hb involved an increase from baseline of 2.5 g/dL. Hb decreased 1.2 g/dL after initiation of the dose delay. An additional dose delay was reported after Hb increased 4.1 g/dL from baseline (the investigator did not report this as an AE after determining it was not a significant change); Hb decreased 0.5 g/dL after the dose delay and restarting treatment. Platelet values for the thrombocytopenia events were found to have decreased from baseline by 47 × 10^9^/L (from 92 × 10^9^/L to 45 × 10^9^/L) and 45 × 10^9^/L (from 84 × 10^9^/L to 39 × 10^9^/L) for each event; after dose delay, restarting sotatercept at 0.3 mg/kg and re-escalating to 0.7 mg/kg, platelet values were found to have increased 77 × 10^9^/L (from 45 × 10^9^/L to 122 × 10^9^/L) and 37 × 10^9^/L (from 39 × 10^9^/L to 76 × 10^9^/L) for each event. No event was considered severe and all participants subsequently re-escalated 0.7 mg/kg per the guideline.

### Cumulative data from primary and extension period up to data cutoff date

For the cumulative period including the primary treatment period and the extension up to the data cutoff date, there were 44 participants (95.7%) with 1 or more AEs and 7 (15.2%) with SAEs with none related to study medication and no discontinuations due to AEs or AEs resulting in death. Overall, the safety results for this cumulative period were consistent with the results for the primary treatment period; the most common AEs were nasopharyngitis (43.5%, 20 participants), epistaxis (26.1%, 12 participants), headache (21.7%, 10 participants), and increased Hb (21.7%, 10 participants). There were no additional telangiectasia AEs in the extension period, and the most frequently reported AEs of interest were bleeding events (37.0%, 17 participants) and immunogenicity and increased Hb (21.7%, 10 participants each) (Supplemental Table 4).

After the data cutoff date, 1 participant died due to gastrointestinal hemorrhage and the event was considered related to study intervention by the investigator. The participant had a diagnosis of idiopathic PAH and was on parenteral prostacyclin and oral ERA treatment as background PAH therapy. Previous worsening of PAH was noted on day 252 of the study. The last administration of sotatercept, which has a half-life 23 days,^18^ was 82 days before an upper gastrointestinal endoscopy confirmed bleeding and 88 days before the participant’s death. The death occurred beyond the 56 days of the safety analysis period following the last dose of the study intervention.

In addition, there were no AEs of pericardial effusion or hypoxemia due to intrapulmonary shunting observed or reported.

### Immunogenicity

Eighteen of 46 participants (39.1%) were positive for sotatercept antidrug antibodies (ADAs). Of these participants, 12 (26.1%) were negative and 6 (13.0%) were positive for neutralizing antibodies. There was no notable effect of ADAs on pharmacokinetics and the effects on key efficacy endpoints were generally comparable across ADA status (Supplemental Table 4). AEs categorized as immunogenicity-related events by predefined search criteria occurred in 9 participants who reported dermatitis, allergic dermatitis, bullous dermatitis, drug eruption, rash, urticaria, and vancomycin infusion reaction.

## Discussion

This open-label, phase 3 study is the first clinical trial to evaluate the safety and efficacy of sotatercept in a study population comprised specifically of Asian participants with PAH. The results of this trial showed that Japanese participants with PAH on stable background therapy achieved clinically important improvements with the addition of sotatercept in measurements of hemodynamics, cardiac function, and exercise capacity as shown by substantial decreases in PVR and NT-proBNP and increases in 6MWD. Further, treatment with sotatercept in this population was associated with a relatively low incidence of serious AEs and the overall safety profile was consistent with previous studies in non-Japanese study populations with no specific findings for Japanese participants (Central Illustration).

**Central Illustration:**
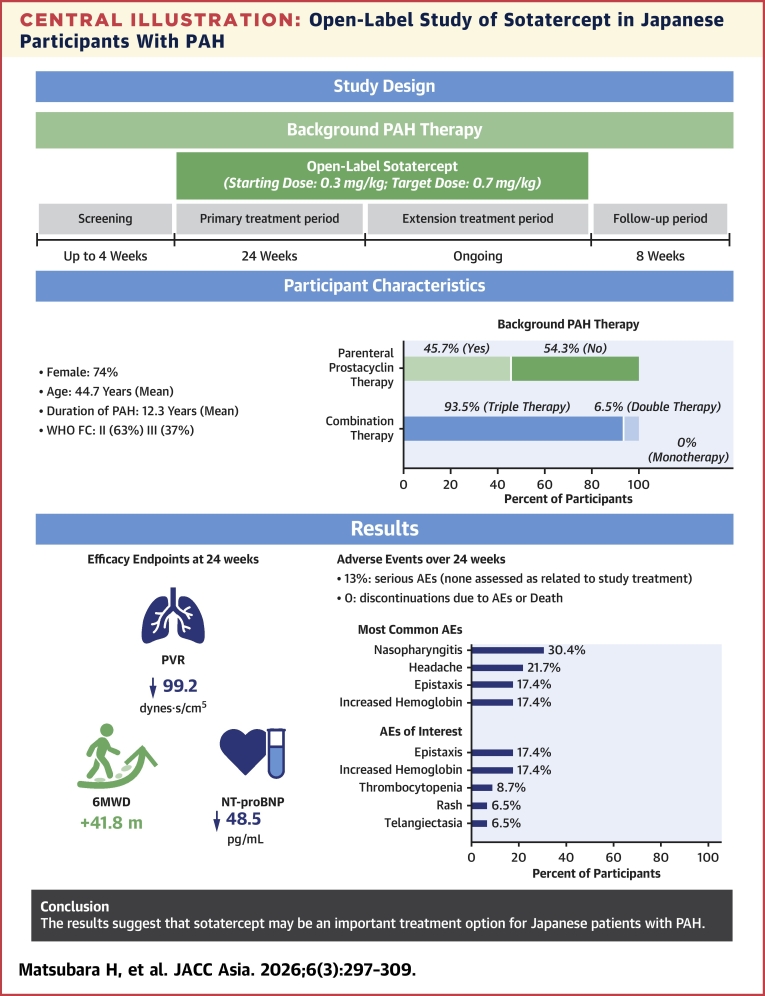
Open-Label Study of Sotatercept in Japanese Participants With PAH In **t**his open-label, single-arm, phase 3 study in adult Japanese participants with pulmonary arterial hypertension (PAH) on stable background therapy, sotatercept treatment over 24 weeks led to improvements in pulmonary vascular resistance (PVR) and 6-minute walk distance (6MWD) with safety consistent with the known safety profile of this treatment. AE = adverse event; FC = functional class; NT-proBNP = N-terminal pro–B-type natriuretic peptide.

The improvements observed in this study are indicative of a positive clinical impact in Japanese PAH patients. The primary efficacy endpoint in this study was change from baseline in PVR at week 24. High PVR is associated with right ventricular dysfunction in PAH and reduction in PVR with treatment leads to improved prognosis and reduced risk.^10^ Further, reduction in PVR is predictive of improved survival in PAH.^9^^,^^10^ Although the improvement in PVR observed in this trial appears to be smaller than those observed in the global phase 3 STELLAR trial,^5^^,^^13^ the severity of disease is evidently lower in this study population compared with the STELLAR trial based on the comparison of baseline PVR levels and the distribution of participants according to WHO functional class.^13^ The mean duration of PAH at baseline was longer than that observed in the STELLAR trial and most participants (ie, more than 90%) were receiving triple therapy in this study whereas it was approximately 60% in the STELLAR trial. Overall, evaluation of baseline characteristics for this study of Japanese PAH participants suggests that this study population had less severely impaired hemodynamics at baseline than those of the STELLAR trial.^13^ Despite this, the benefit observed with use of sotatercept in each study population was relatively similar with a –29.9% (95% CI: –34.0% to –25.5%) geometric mean percent change from baseline in PVR at week 24 in the STELLAR trial and –21.3% (95% CI: –26.7% to –15.5%) in this study. The same discussion also applies to the PULSAR trial,14 in which the participant population had baseline characteristics very similar to the STELLAR trial and the study design was similar with change from baseline to week 24 in PVR as the primary endpoint, but with the key difference of the inclusion of a 0.3 mg/kg arm. The baseline characteristics profile in this study appears consistent with the overall Japanese PAH population, which is evident when these data are compared with the demographics observed in the Japan Pulmonary Hypertension Registry (JAPHR), a registry with a larger patient sample (N = 631) and therefore likely more representative of the PAH patient population in Japan. In the JAPHR, PAH patients had a mean age at diagnosis of 52.7 years, with a predominance of female patients (79.4%). Disease severity according to baseline clinical endpoint levels (stated above) were consistent with what was observed in this study. A notable difference is that in JAPHR, 39% of participants were receiving triple therapy, indicating that the participants in this phase 3 study were receiving more aggressive therapy than the patients in the JAPHR registry.^7^

The addition of sotatercept to standard-of-care treatments for PAH in this study was generally well tolerated in a patient population that typically exhibits elevated levels of morbidity. The tolerability of sotatercept was evident during the cumulative 24-week primary treatment period and the extension, which, as of the cutoff date, was a total mean duration of exposure of 228.6 ± 34.4 days. The incidence of serious AEs was relatively low and none of the serious AEs were deemed to be related to study medication. There were no AEs leading to discontinuation of treatment up to the data cutoff date. AEs associated with known effects of sotatercept on hematologic parameters were of special interest in this study. Telangiectasia AEs and thrombocytopenia occurred with relatively low frequency in this study. Increased Hb and bleeding events occurred at levels consistent with previous clinical studies.^13^^,^^14^ AEs during the cumulative primary and extension periods up to the cutoff date were clinically manageable through protocol-specified dose modification. Dose delays were implemented in only 3 participants who experienced increased Hb levels or thrombocytopenia with no discontinuation of treatment. Additionally, approximately 40% of participants were found to be positive for ADAs, but no impact on safety and efficacy was observed.

There were no deaths or drug-related serious bleeding events during the cumulative period before the cutoff date. However, there was a death reported after the cutoff date resulting from gastrointestinal hemorrhage deemed related to study medication. Although this event occurred after the cutoff date, it is noted in this report as bleeding AEs are an important consideration for prescribing physicians and patients.^19^ Serious bleeding is more likely in patients on background prostacyclin therapy and/or antithrombotic agents or those with low platelet counts.^19^ The participant who died in this study was on prostacyclin infusion and oral ERA treatment as background PAH therapy. Based on the overall safety reporting from clinical studies of sotatercept, the signs and symptoms of blood loss, particularly in patients with prostacyclin and antithrombotic treatment, should be monitored.

### Study limitations

The open-label design may introduce bias, as both participants and investigators were aware of the treatment being administered. Additionally, the absence of a comparator group limits objective assessment of the efficacy and safety of sotatercept relative to a placebo. Further, the relatively small sample size may restrict the generalizability of the findings and limits the ability to detect rare adverse events such as pericardial effusion or hypoxemia due to right-to-left intrapulmonary shunting which were recently described in case reports.^20^, ^21^, ^22^, ^23^ Although these events were not observed or reported in this study, the study was not powered to detect these rare events and conclusive assessment of risk in Japanese patients with PAH requires further experience with greater numbers of patients treated with sotatercept. Another important consideration is that the participant population in this study reflects the aggressive treatment standards for PAH in Japan; this may represent a limitation for the interpretation of results in the context of patients with a more advanced state of disease or in areas of the world with less stringent therapeutic guidelines. In addition, this is an ongoing study and longer-term data from this study will be forthcoming to assess long-term efficacy and safety including potential impact of immunogenicity. Finally, self-administration of sotatercept at home during the extension period is of clinical interest, but at the time of the data cutoff date, the number of participants who were self-administering at home was limited. Therefore, this will be described in a future publication when a fuller dataset is available. Nevertheless, these data provide evidence of meaningful outcomes in Japanese patients with PAH using sotatercept, which provides important information for this patient population and prescribing physicians.

## Conclusions

The addition of sotatercept to standard-of-care background therapy for PAH suggests a clinically meaningful improvement in PVR, exercise tolerance, WHO functional class status, and NT-proBNP levels. Further, the AEs in this study were generally manageable and resulted in no new safety findings from what has previously been reported in clinical studies. These results suggest the potential of sotatercept as a promising new treatment option for Japanese patients with PAH that would substantially improve overall outcomes for this patient population.

### Data Availability

The data sharing policy, including restrictions, of Merck Sharp and Dohme LLC, a subsidiary of Merck & Co, Inc, Rahway, New Jersey, USA (MSD), is available at https://trialstransparency.msdclinicaltrials.com/policies-perspectives.aspx. Requests for access to the clinical study data can be submitted via email to the Data Access mailbox (dataaccess@msd.com).

## Funding Support and Author Disclosures

Dr Matsubara has received consulting fees from Bayer, Janssen Pharmaceutical KK, MSD KK, and Mochida Pharmaceutical Co Ltd has received speakers fees from Bayer, Janssen Pharmaceutical KK, MSD KK, Mochida, AOP-health, Kaneka Medix Co, Nippon Shinyaku Co Ltd, Nipro, and United Therapeutics; and has received research funds from Nippon Shinyaku Co Ltd. Dr Tanabe has received personal fees from MSD KK, Nippon Shinyaku, and Janssen Pharmaceutical KK. Dr Ogo has received speakers fees from Janssen Pharmaceutical KK, Nippon Shinyaku Co Ltd, Bayer Yakuhin Ltd, Pfizer Japan Inc, Mochida Pharmaceutical Co Ltd, and MSD KK. Dr Abe has received grants from Konica Minolta and Daiichi-Sankyo. Dr Inami has received personal fees from MSD KK and Janssen Pharmaceutical KK. Dr Maeda, Dr Arano, Mr Shirakawa, and Mr Sakai are employees of MSD K.K., Tokyo, Japan and may own stock and/or hold stock options in Merck & Co., Inc., Rahway, NJ, USA. Dr Cornell is an employee of Merck Sharp and Dohme LLC, a subsidiary of Merck & Co Inc, Rahway, New Jersey, USA, who may own stock and/or hold stock options in Merck & Co Inc, Rahway, New Jersey, USA.
