# Supplementary material for: Phase 3, Open-Label Multicenter Study of Sotatercept in Japanese Participants With Pulmonary Arterial Hypertension
Source: JACC Asia. 2026 Jan 31;6(3):297–309. doi: 10.1016/j.jacasi.2025.12.009 (PMC12959282; doi:10.1016/j.jacasi.2025.12.009)

**Supplement**

**Supplemental Table 1 –
Sotatercept study 020 investigators (Primary investigators and study sites)**

| Primary Investigator | Study site | Ethics Committees and Institutional Review Boards |
| --- | --- | --- |
| Ichizo Tsujino | Hokkaido University Hospital | Hokkaido University Hospital Institutional  Review Board  Kita 14, Nishi 5, Kita-ku  Sapporo, Hokkaido 060-8648  Japan |
| Nobuhiro Yaoita | Tohoku University Hospital | Tohoku University Hospital Institutional  Review Board  1-1, Seiryomachi, Aoba-ku  Sendai, Miyagi 980-8574  Japan |
| Toshihiko Sugiura | Chiba University Hospital | Chiba University Hospital Institutional  Review Board  1-8-1, Inohana, Chuo-Ku  Chiba, Chiba 260-8677  Japan |
| Nobuhiro Tanabe | Chibaken Saiseikai Narashino Hospital | Chibaken Saiseikai Narashino Hospital  Institutional Review Board  1-1-1 Izumicho  Narashino, Chiba 275-8580  Japan |
| Takumi Inami | Kyorin University Hospital | Institutional Review Board of Kyorin  University Hospital  6-20-2 Shinkawa  Mitaka-shi, Tokyo 181-8611  Japan |
| Masaru Hatano | The University of Tokyo Hospital | The Institutional Review Board, The University of Tokyo Hospital  7-3-1, Hongo  Bunkyo, Tokyo 113-8655  Japan |
| Takahiro Hiraide | Keio University Hospital | Keio University Hospital Institutional  Review Board  35 Shinanomachi,  Shinjuku, Tokyo 160-8582  Japan |
| Yuichi Tamura | International University of Health and Welfare, Mita Hospital | International University of Health and  Welfare - Institutional Review Board  1-24-1, Minamiaoyama  Minato, Tokyo 107-0062  Japan |
| Shiro Adachi | Nagoya University Hospital | Nagoya University Hospital Institutional  Review Board  65, Tsurumai-cho, Showa-ku  Nagoya, Aichi 466-8560  Japan |
| Takeshi Ogo | National Cerebral and Cardiovascular Center | National Cerebral and Cardiovascular  Center Institutional Review Board  6-1, Kishibe-Shimmachi  Suita, Osaka 564-8565  Japan |
| Yu Taniguchi | Kobe University Hospital | Kobe University Hospital Institutional  Review Board  7-5-2, kusunoki-cho, chuo-ku  Kobe, Hyogo 650-0017  Japan |
| Hiromi Matsubara | National Hospital Organization Okayama Medical Center | National Hospital Organization Okayama  Medical Center Institutional Review Board  1711-1 Tamasu Kita-ku  Okayama, Okayama 701-1192  Japan |
| Nobuhiro Tahara | Kurume University hospital | Kurume University Institutional Review  Board  67 Asahi-machi  Kurume, Fukuoka 830-0011  Japan |
| Kohtaro Abe | Kyushu University Hospital | Kyushu University Hospital Institutional  Review Board  3-1-1, Maidashi, Higashi-ku  Fukuoka, Fukuoka 812-8582  Japan |
| Hiroshi Watanabe | Hamamatsu University Hospital | Institutional Review Board of Hamamatsu  University Hospital  1-20-1 Handayama, Chuo-ku,  Hamamatsu, Shizuoka 431-3192  Japan |
| Yoshihiro Dohi | Kure Kyosai Hospital | Federation of National Public Services  and Affiliated Personnel Mutual Aid  Associations Kure Kyosai Hospital  Institutional Review Board  2-3-28, Nishi-Chuo, Kure-shi, Hiroshima-ken  737-8505  Japan |
| Akiyoshi Hashimoto | Sapporo Medical University Hospital | Sapporo Medical University Hospital  Institutional Review Board  Minami 1-jo Nishi 16-chome Chuo-ku  Sapporo, Hokkaido 060-8543  Japan |

**Supplemental Table 2 - Change From Baseline in PVR, 6MWD and NT-proBNP at Week 24 in the FAS Population (Post-Hoc Analysis - Mixed Effects Quantile Regression)**

| Endpoint | Median (95% CI) |
| --- | --- |
| Change from baseline in PVR (dynes*sec/cm^5^) at Week 24 | -115.7 (-155.8, -75.6) |
| Change from baseline in 6MWD (m) at Week 24 | 41.6 (20.9, 62.2) |
| Change from baseline in NT-proBNP (pg/mL) at Week 24 | -42.5 (-90.5, 5.5) |
| Based on a mixed effects quantile regression model with age, sex, baseline value, WHO functional class at baseline and infusion prostacyclin use at baseline as fixed effect covariates, and study center as a random effect. | |

**Supplemental Table 3 - Change From Baseline in Hemodynamic Parameters at Week 24 in the FAS Population**

| **Treatment** | **Baseline** | | | **Week 24** | | | **Change from baseline**  **at Week 24** |
| --- | --- | --- | --- | --- | --- | --- | --- |
|  | **N** | **Mean** | **(SD)** | **N** | **Mean** | **(SD)** | **Mean (95% CI)** |
| **Cardiac Output (L/min)** | 46 | 4.8 | 1.1 | 46 | 4.8 | 1.7 | -0.1 (-0.4, 0.3) |
| **Mean PAWP (mmHg)** | 46 | 8.1 | 3.0 | 46 | 7.9 | 3.1 | -0.2 (-1.0, 0.6) |
| **RAP (mmHg)** | 46 | 5.2 | 2.9 | 46 | 4.2 | 3.1 | -1.0 (-1.9, -0.2) |
| **SvO2 (%)** | 46 | 70.5 | 5.3 | 46 | 71.1 | 6.8 | 0.6 (-0.9, 2.2) |
| **Mean PAP (mmHg)** | **N** | **Median** | **Range** | **N** | **Median** | **Range** | **Estimate (95% CI)** |
|  | 46 | 40.5 | [28.0, 68.0] | 46 | 31.5 | [22.0, 48.0] | -7.5 (-9.5, -6.0) |
| N = number of participants with data.  The primary approach was to exclude the data after addition, increase or substitute of background PAH therapy, but no participants modified the background PAH therapy during the Primary treatment period and thus no data were excluded.  Estimate (95% CI) for change from baseline in Mean PAP based on the Hodges-Lehmann method  Data Cutoff: 12-Mar-2024. | | | | | | | |

**Supplemental Table 4 – Cumulative Safety and Tolerability (Primary Treatment Period + Extension Period up to Data Cutoff Date)**

|  | Sotatercept | |
| --- | --- | --- |
|  | n | (%) |
| Participants in population | 46 | |
| **Summary of AEs in Primary Treatment Period** | | |
| ≥1 AE | 44 | (95.7) |
| Serious AEs | 7 | (15.2) |
| Discontinuations due to AEs | 0 | (0.0) |
| Deaths | 0 | (0.0) |
| Drug-related AEs | 29 | (63.0) |
| Drug-related Serious AEs | 0 | (0.0) |
| **Most Common AEs (≥10%)** | | |
| Nasopharyngitis | 20 | (43.5) |
| Hemoglobin Increased | 10 | (21.7) |
| Headache | 10 | (21.7) |
| Epistaxis | 12 | (26.1) |
| **AEs of Interest (≥5%)** | | |
| Telangiectasia | 3 | (6.5) |
| Epistaxis | 12 | (26.1) |
| Rash | 3 | (6.5) |
| Hemoglobin Increased | 10 | (21.7) |
| Thrombocytopenia *(reported as thrombocytopenia or platelet count decreased)* | 4 | (8.7) |

**Supplemental Table 5 - Change from baseline in PVR, 6MWD, and NT-proBNP by ADA status at Week 24 in the FAS population**

| **ADA status** | **N** | **Mean** | **(SD)** | **Median** | **IQR** |
| --- | --- | --- | --- | --- | --- |
| **PVR (dynes*sec/cm^5^), Week 24** | | | | | |
| Negative | 28 | -128.0 | (143.2) | -108.0 | [-172.0, -52.4] |
| Positive, NAb negative | 12 | -85.1 | (100.6) | -69.2 | [-155.6, -48.0] |
| Positive, NAb positive | 6 | -107.2 | (171.0) | -50.4 | [-64.0, -36.8] |
|  |  |  |  |  |  |
| **6MWD (m), last post-baseline measurement** | | | | | |
| Negative | 28 | 40.3 | (57.1) | 45.3 | [10.8, 72.8] |
| Positive, NAb negative | 12 | 55.3 | (48.6) | 48.0 | [17.3, 93.8] |
| Positive, NAb positive | 6 | 33.6 | (27.1) | 33.8 | [14.0, 62.0] |
|  |  |  |  |  |  |
| **NT-proBNP (pg/mL) , last post-baseline measurement** | | | | | |
| Negative | 28 | -78.1 | (164.7) | -41.8 | [-103.0, 0.0] |
| Positive, NAb negative | 12 | -75.4 | (316.9) | -30.3 | [-60.3, 13.5] |
| Positive, NAb positive | 6 | -159.6 | (295.5) | -37.0 | [-128.5, 0.0] |

ADA positive includes both treatment-emergent positive and treatment-boosted positive

N=number of participants with a valid change from baseline result

The primary approach was to exclude the data after addition, increase or substitute of background PAH therapy, but no

participants modified the background PAH therapy during the Primary treatment period and thus no data were excluded

**Supplemental Figure 1 - Protocol for Dose Modifications**

**A. Target Dose Escalation** **
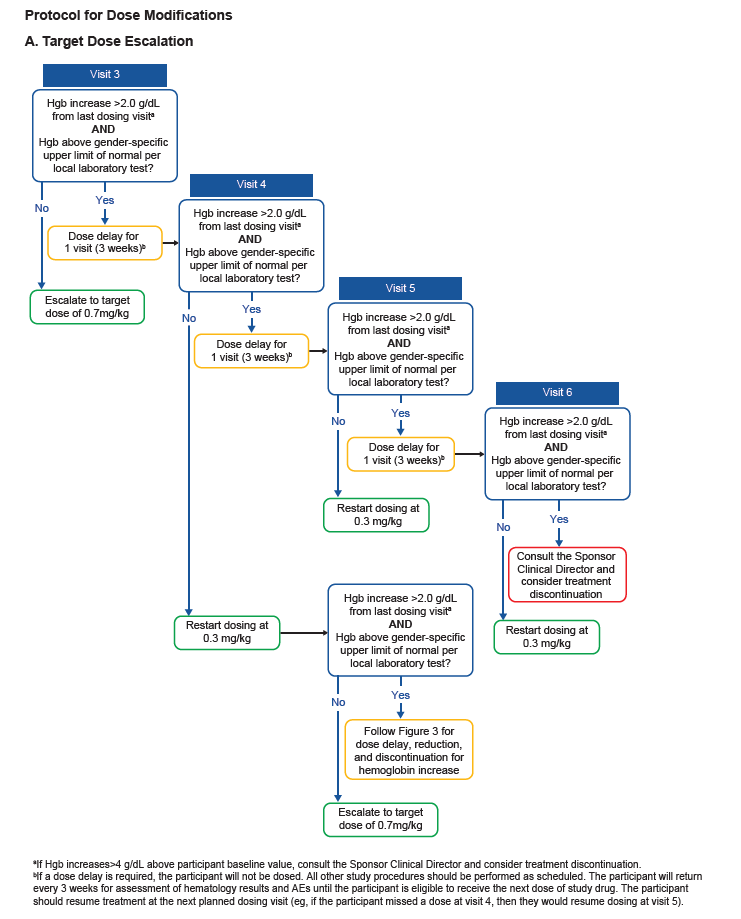
**

**B. Dose Modification Due to Hemoglobin Increase**

**
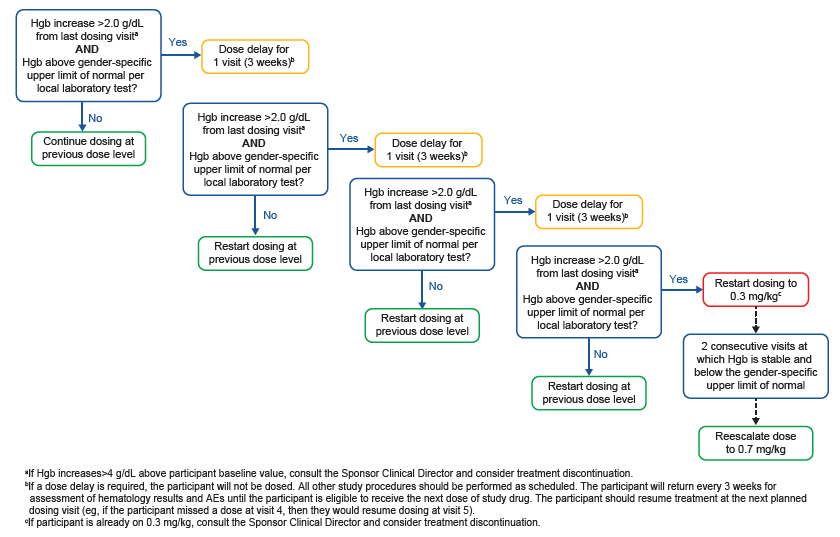
**

**C. Dose Modification due to Low Platelet Count**

**
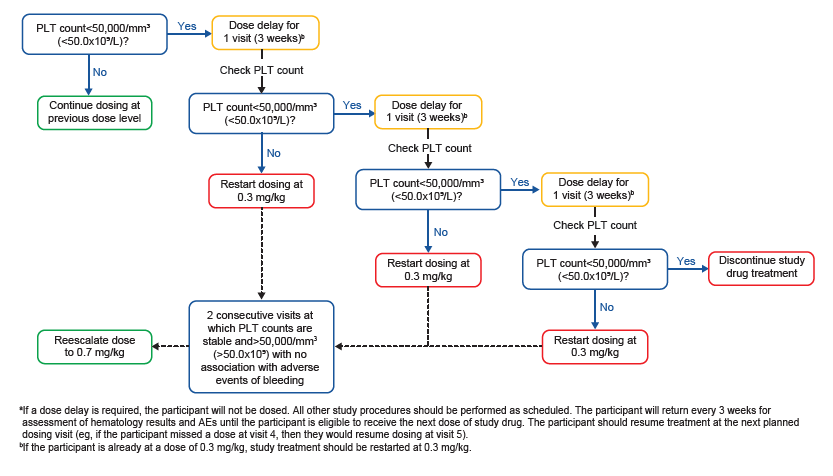
**

**Supplemental Figure 2 - Forest Plot for the Subgroup Analysis of Change from Baseline in PVR (dynes*sec/cm^5^) at Week 24 (FAS Population)**

**
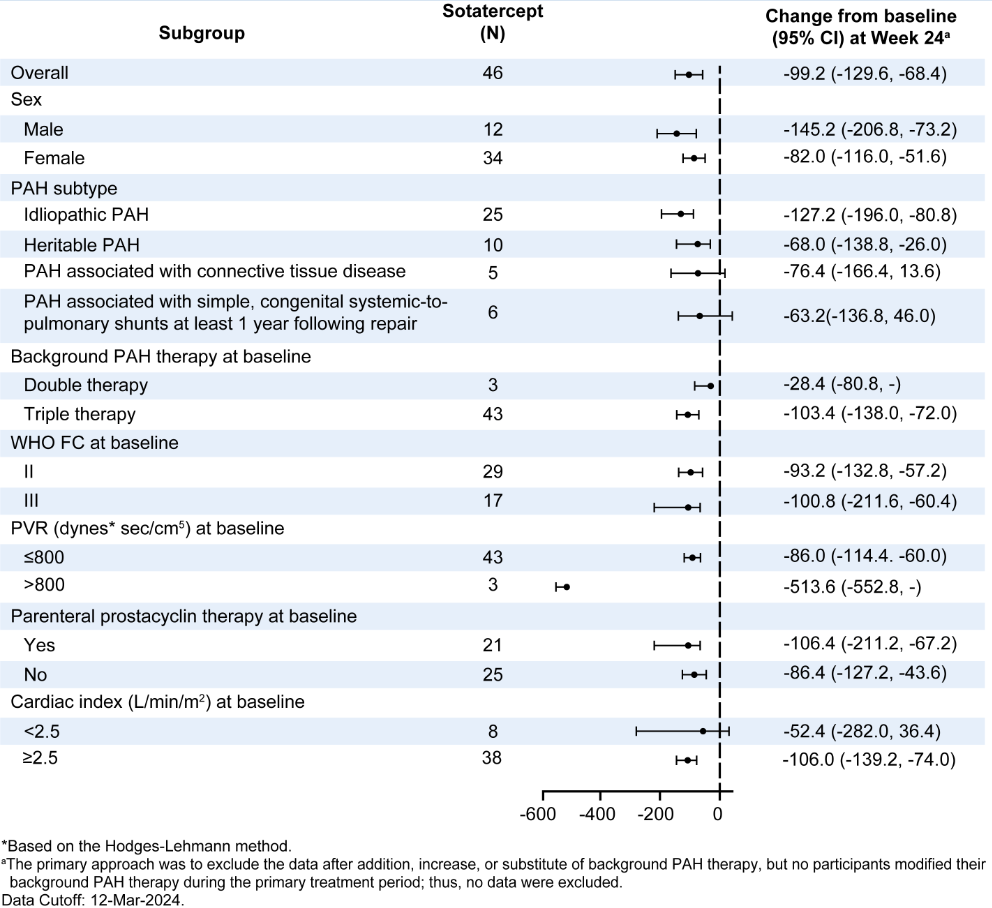
**

**Supplemental Figure 3 - Forest Plot for the Subgroup Analysis of Change from Baseline in 6MWD (m) at Week 24 (FAS Population)**


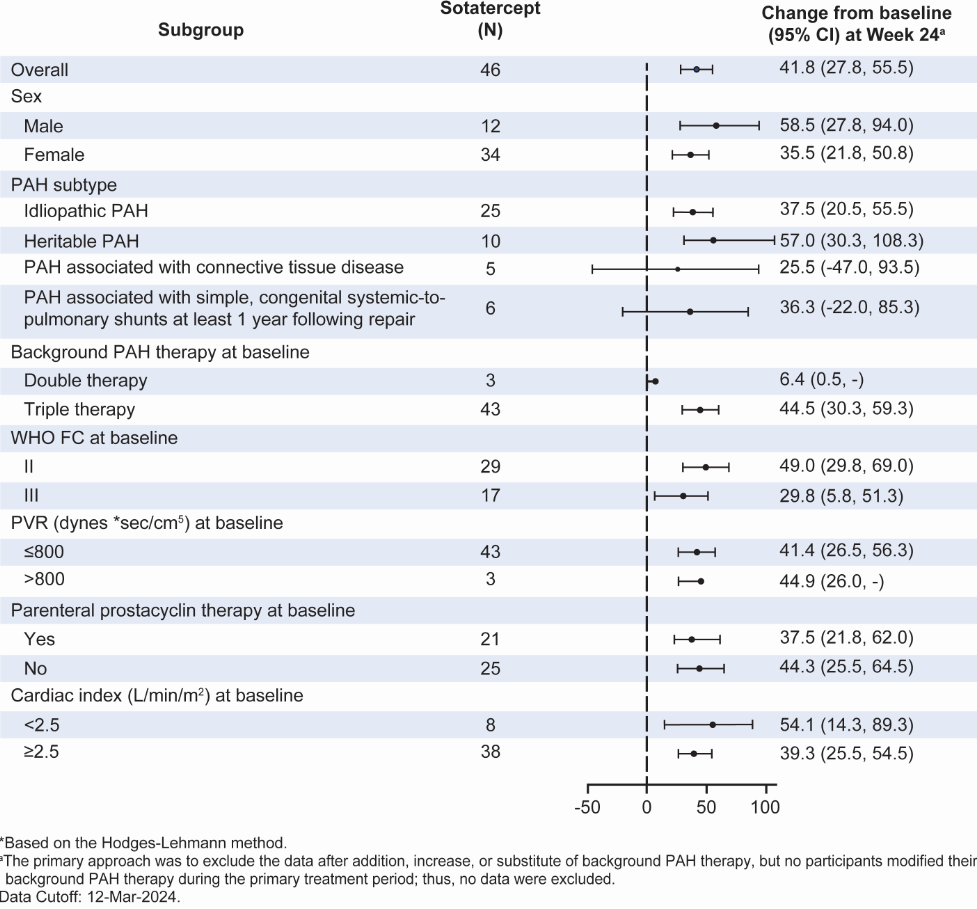


**Supplemental Figure 4 - Forest Plot for the Subgroup Analysis of Change from Baseline in NT-proBNP (pg/mL) at Week 24 (FAS Population)**


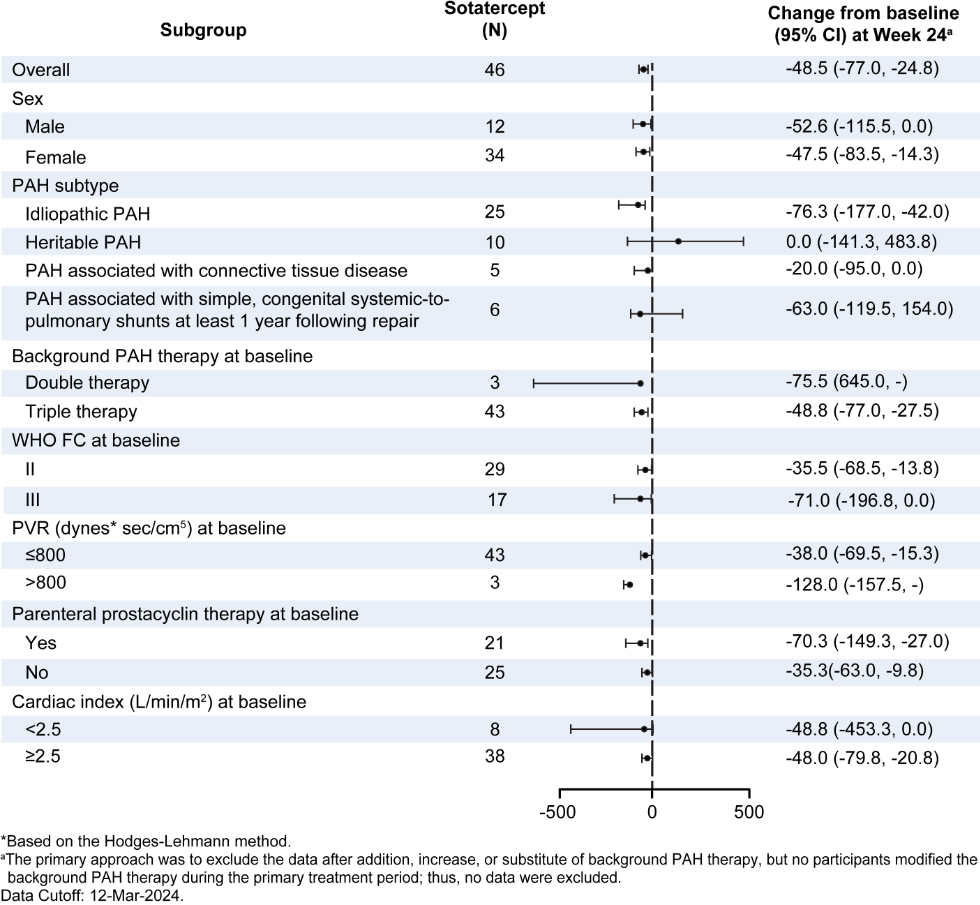


**Supplemental Figure 5 - Kaplan-Meier Plot of Time to First Clinical Worsening of Death (FAS Population)**


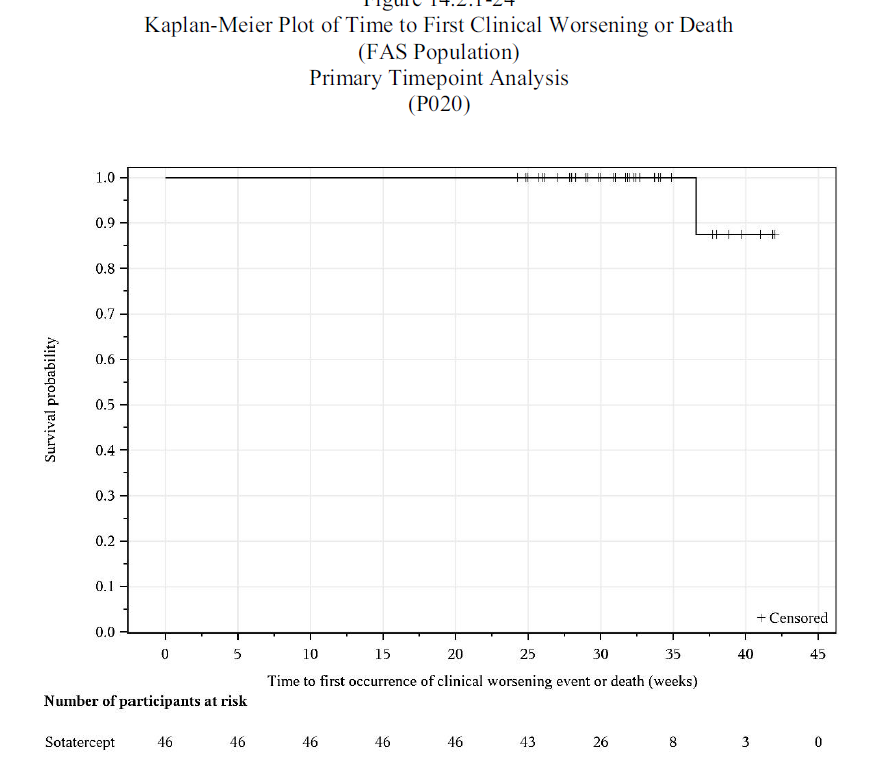

Supplement: Supplemental Material [file mmc1.docx]
